# The Book World of Medicine and Science

**Published:** 1896-03-14

**Authors:** 


### THE BOOK WORLD OF MEDICINE AND SCIENCE.

THE MEDICAL DIGEST: Appendix, including the years 1891-2 3-4, and to August, 1895. By RICHARD NEALE, M.D. Lond. (London: Ledger, Smith, and Co., 1895.)

The "Medical Digest" is too well known to require fresh praise, and its utility is greatly increased by the issue of this appendix, bringing it practically up to date. The service rendered by Dr. Neale to all whose work leads them to consult the endless volumes of periodical literature, in which the results of so much patient medical investigation would, but for his labours, lie buried and lost, is incalculable. The "Digest" is indeed the key by which alone the contents of medical libraries are revealed to many inquirers. Nevertheless, we have a little quarrel with this appendix. It is so arranged that it is almost useless, except when read in conjunction with the edition of 1890. In a library no doubt that is the proper way to use it, but we should imagine that there must be hundreds of medical men who would be glad to have an analysis of the past five years of medical literature, who yet hesitate to buy the larger work, and certainly for ordinary daily reference would not look through two indices for every subject wanted. A five-yearly digest like this is a most valuable work, but to be really useful it should be so indexed as to be complete in itself.

THE TREATMENT OF PULMONARY CONSUMPTION. (Lewis's Practical Series.) By VINCENT DORMER HARRIS, M.D., F.R.C.P., and EDWIN CLIFFORD BEALE, M.B., F.R.C.P. (London: H. K. Lewis. 1895. Price 10s. 6d.)

Consumption is so common a disease, and the treatment of its various phases forms so large a part of the work of most medical men, that the appearance of such a manual as this, giving in handy and accessible form the results of modern experience on the subject, is very welcome, and all the more so from the fact that it shows evidence throughout that the conclusions arrived at are the result of an extensive personal contact with the disease. After a short sketch of the history and pathology of phthisis, and a description of the methods of its arrest and cure, its causes and its prophylactic treatment, the main subject is entered upon, viz., that of treatment. While admitting the advantages of fruit farming and cattle ranching in Australia and America as a mode of life likely to be prophylactic against consumption, a caution is given that phthisis is not altogether a matter of climate, that success in business is no more assured in sunny climes than in damp or cloudy ones, and that the disappointment of failure in business has again and again led to failure in health and to the active development of tubercular disease, notwithstanding the perfection of the climatic surroundings. This is a point that has been far too much neglected. It is not so much actual want that leads to phthisis as penury and the anxious carefulness entailed by having to live below the accustomed level—so much are we creatures of habit and custom. The question of treatment is considered under the heads of early phthisis, the progressive disease, the quiescent or cured disease, modes of dealing with symptoms and complications, and chapters are given on diet, special forms of treatment, and on the various climates which are most useful to those who are affected or threatened by the malady. Many useful formulæ are interspersed in the text, the directions in regard to climate are full of common sense, and the chapter on special treatment is a useful review of the various new methods that have been advocated during the last few years. We have been much pleased with the book, which fittingly forms part of a "Practical Series."

PEDIATRICS. A NEW JOURNAL. (New York: Van Publishing Company.)

"Pediatrics" is a new journal devoted to the diseases of children, which is issued in semi-monthly parts, with illustrations, and the price is 8s. a year. The editor of this new journal is Dr. Carpenter, senior physician to out-patients Evelina Hospital for Children, and the editorial staff includes Dr. Jacobi, of New York, Dr. Henry Ling Taylor (New York), Mr. F. S. Eve (London), and a number of other well-known specialists in the various branches of pediatrics. The first number, which we have before us, contains an original article on infant feeding, by Dr. Jacobi. It is an excellent exposition of his individual views on this vexed question, and contains some suggestive and useful hints for the establishment of infant dietetics on a scientific basis. Artificial feeding of children should be carried out as far as possible on Dr. Rolch's plan, with the establishment of milk laboratories in the large towns. Thus, to suit the various idiosyncrasies of different children, modified milk might be ordered containing different proportions of the several constituents. For example, for a healthy baby of four months the food prescription would run as follows: Fat, 4; milk sugar, 7; albuminoids, 1·50. Put up eight tubes each containing 4 ounces, with lime water 5 per cent. Pasteurise (75 degrees C.) for twenty minutes. This article of Dr. Jacobi opens the ball with considerable *éclat*, and we can only hope that its high standard of excellence will be maintained in subsequent numbers. Other articles are by Drs. Fruitnight and Brown on a case of cretinism, with illustrations; the Japanese ice bag, by Dr. Phelps; and a case of fibroid phthisis by Dr. Sutherland; which with practical, editorial, and other notes, together with notices on new books and abstracts from other papers, go to make up an excellent number.

THE GARDEN ORACLE AND ILLUSTRATED FLORICULTURAL YEAR BOOK. By the Editor of the "Gardeners' Magazine." (The "Gardeners' Magazine" Office, 4, Ave Maria Lane, E.C. Price 1s.)

This useful handbook deserves to meet with approval from all members of the gardening world. Both the most inexperienced of amateurs and the most ambitious of professionals will find it full of excellent hints, and to the former class the illustrations will be most acceptable.

